# Supplementary material for: C53 Interacting with UFM1-Protein Ligase 1 Regulates Microtubule Nucleation in Response to ER Stress
Source: Cells. 2022 Feb 5;11(3):555. doi: 10.3390/cells11030555 (PMC8834445; doi:10.3390/cells11030555)
Supplement: Supplementary file 1 [file cells-11-00555-s001.zip › cells-1469163-supplementary.pdf]

Text S1 Features of in-house written macro for processing images from ER area quantification experiments

We developed a macro for the measuring of ER area (images collected either from living cells or from fixed samples) written in ImageJ Macro Language in Fiji. ER was marked by ER-Tracker Green or by immunostaining for calnexin, cell boundaries were delineated by fluorochrome-tagged WGA, and nuclei were labeled by Hoechst 33342 or DAPI. Firstly, we manually draw an ROI exactly around the whole cell. Then the macro cleared the image stack outside the cell from any undesired signal, e.g., background, neighboring touching cells. Afterward, by using nuclear signal and consecutive segmentation of a nucleus by automatic thresholding (Li method), the nucleus was removed from the cell since its intensities would negatively affect the measurements. Then maximum intensity projection was applied to accumulate ER signals from the entire cell into one picture. By automatic thresholding (Li method), we converted the area of the ER signal to a mask. By measuring the mask area, we got the area size of ER in the cell. Subsequently, we inverted this mask and obtained the area without the ER signal. We measured this area as well. Finally, we calculated ER area coefficient (area occupied by ER/area free of ER). ER coefficients enabled us to describe differences among cells that may differ by various densities of the ER markers.

Table S1 Sequences of primers used for real time qRT-PCR analysis of human genes

Name	Sequence	Amplicon length
CANX, fwd	5′- GAAGAGGCCACAAAACCCGA -3′	
CANX, rev	5′- GCCTCCCATTCTCCATCCA -3′	112bp
P4HB, fwd	5′- AAAACGTCTTTGTGGAGTTC -3′	
P4HB, rev	5′- TACGTCTCTCTCCCAGTTTATC -3′	80bp
DDIT3, fwd	5′- CTTTTCCAGACTGATCCAAC -3′	
DDIT3, rev	5′- GATTCTTCCTCTTCATTTCCAG -3′	172bp
HSPA5, fwd	5′- TCTATGAAGGTGAAAGACCC -3′	
HSPA5, rev	5′- TCTCAAAGGTGACTTCAATC -3′	113bp
ERN1, fwd	5′- GAATAGAAAAGGAATCCCTGG -3′	
ERN1, rev	5′- TTCTTATTTCTCATGGCTCG -3′	180bp
ATF6A, fwd	5′- AATATATGCTAGGGTTAGAGGC-3′	
ATF6A, rev	5′- TTCTCTGACACAACTTCATC-3′	111bp
PPIA, fwd	5′- GAGCACTGGGGAGAAAGGAT -3′	
PPIA, rev	5′- CTTGCCATCCAGCCACTCAG -3′	258bp

Table S2 **Mass spectrometry identification of E3 UFM1-protein ligase 1 (UFL1)**

Measured mass [M+H]	Computed mass [M+H]	Error (ppm)	Peptide sequence	Peptide position
1292.619	1292.605	11	MoxADAWEEIRR	1 - 10
820.454	820.431	28	LAADFQR	11 - 17
1037.488	1037.501	13	AQFAESTQR	18 - 26
2369.293	2369.271	9	QLEVVHTLDGKEYITPAQISK	44 - 64
2305.286	2305.251	15	VNIVDLQQVINVDLTHIESR	77 - 96
1808.942	1808.918	13	AYDLPGDFLTQALTQR	145 - 160
1279.699	1279.675	19	IINGHLDLDNR	164 - 174
1209.630	1209.663	27	GVIFTEAFVAR	175 - 185
2317.226	2317.171	24	YGFQEQLLYSVLEDLVSTGR	210 - 229
1166.597	1166.620	20	AVFVPDIYSR	242 – 251
1373.633	1373.648	11	TQSTWVDSFFR	252 - 262
1412.713	1412.681	23	QNGYLEFDALSR	263 - 274
1445.695	1445.673	15	FITDCcamTGLFSER	368 - 379
1521.752	1521.791	26	NNPVHLITEEDLK	390 - 402
2049.112	2049.061	25	TIKDLQEEVSNLYNNIR	535 - 551
1706.800	1706.834	20	DLQEEVSNLYNNIR	538 - 551
941.514	941.532	19	QILFQHR	662 - 668

Mox: oxidized methionine; Ccam: carbamidomethylated cysteine

1 **MADAWEEIRR LAADFQRAQF AESTQR**LSER NCIEIVNKLI SQK**QLEVVHT LDGKEYITPA**

61 **QISK**EMRDEL HVRGGR**VNIV DLQQVINVDL THIESR**VSDI IKSEKHVQMV LGQLIDENYL

121 DQLSEEVNDK LQESGQVTVS ELCK**AYDLPG DFLTQALTQR** LGR**IINGHLD LDNRGVIFTE**

181 **AFVAR**HKARI RGLFSAITRP TPVNSLVSK**Y GFQEQLLYSV LEDLVSTGR**L RGTVVGGRQD

241 K**AVFVPDIYS RTQSTWVDSF FRQNGYLEFD ALSR**LGIPDA VNYIKKRYKN TQLLFLKATC

301 VGQGLVDQVE ASVEEAISSG TWVDISPLLP SSLSVEDAAM LLQQVMRPFG KLASAIVFSD

361 TVVVSEK**FIT DCTGLFSER**M HQKAEKEMK**N NPVHLITEED LK**QISILESV NTSKKDKKDE

421 RRKKATEGSG SVRGGGGGNA REYKIKKTKK KGRKDEDSDD ESQSSHGGKK KPDITFMFQD

481 EIEDCLRKHI QDAPEEFISE LAEYLIKPLN KMYLEVVRSV FMSSTSASGT GRKR**TIKDLQ**

541 **EEVSNLYNNI R**LFEKGMKYF ADDTQTALTK HLLKTVCTDI TNLMFNFLAS DFLMAVEEPA

601 AITSDIRKKI LSKLTEETKV ALTKLHNSLN EKSIEDFLSC LDSATEACDI MVKKGDKKRE

661 R**QILFQH**RQA LCEQLKVTED PALILHLTAV LLFQLSTHSM LHAPGRCVPQ IIAFLHSKIP

721 EDQHTLLVKY QGLVVKQLVS QNKKTGQGED PSSDELDKEQ HDVTNATRKE LQELSLSIKD

781 LVLKSRKSSV TEE

Amino acids of the identified peptides in the mouse E3 UFM1-protein ligase 1 (Uniprot identifier Q8CCJ3) are indicated in bold and underlined (bottom part of the table). The matched peptides cover 26% of the protein sequence.

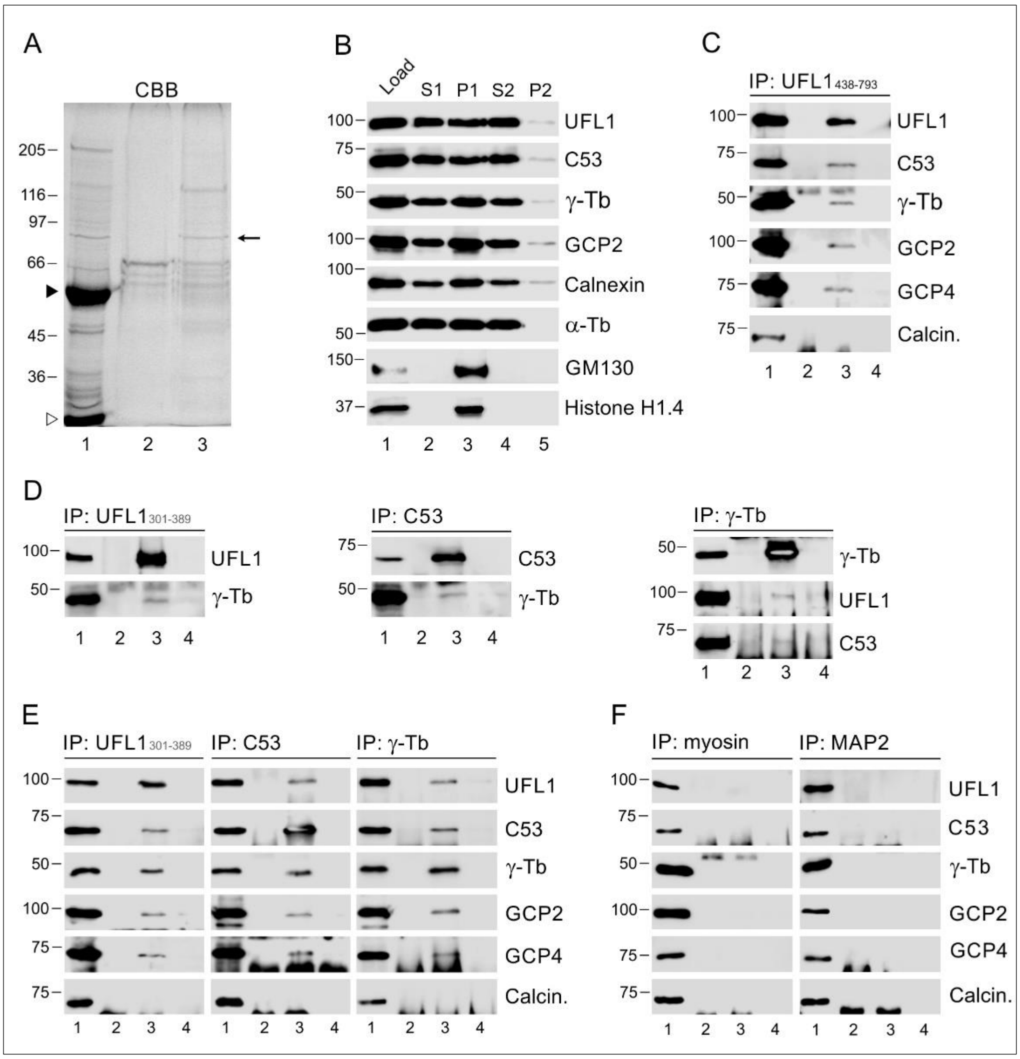

Figure S1. UFL1 and C53 are associated with γTuRC proteins. Controls for immunoprecipitation experiments. (**A**) Coomassie Brilliant Blue G-250 staining of proteins associated with membrane-bound γ-tubulin. Proteins precipitated from the microsomal fraction of differentiated P19 cells with anti-peptide mAb to γ-tubulin TU-31 (*lane 1*), the bound proteins eluted with negative control peptide (*lane 2*), and the bound proteins eluted with immunizing peptide (*lane 3*). Black and empty arrowheads denote immunoglobulin heavy and light chains, respectively. Arrow indicates protein subjected to MALDI/MS fingerprint analysis. (**B-D**) Membrane-bound UFL1 and C53 interact with γTuRC proteins. (**B**) Relative distribution of proteins in fractions after differential centrifugation of the U2OS cell homogenate. Cell fractions were prepared as described in the Materials and Methods section. Cell homogenate (*lane 1*), supernatant S1 (*lane 2*), pellet P1 (*lane 3*), supernatant S2 (*lane 4*), pellet P2 (*lane 5*). To compare the relative distribution of proteins, pelleted material was resuspended in a volume equal to the corresponding supernatant. Blots were probed with Abs to UFL1, C53, γ-tubulin (γ-Tb), GCP2, calnexin, α-tubulin (α-Tb), GM130, and Histone H1.4. (**C-D**) Immunoprecipitation experiments. Extracts from the membranous fraction (P2) of U2OS (**C**) or T98G (**D**) cells were precipitated with immobilized Abs specific to UFL1$_{438-793}$ (**C**) or UFL1$_{301-389}$, C53, and γ-tubulin (**D**). Blots were probed with Abs to UFL1, C53, γ-tubulin (γ-Tb), GCP2, GCP4, and calcineurin (Calcin.; negative control). Load (*lane 1*), immobilized Abs without cell extracts (*lane 2*), precipitated proteins (*lane 3*), and Ab-free carriers incubated with cell extracts (*lane 4*). (**E**) UFL1 and C53 in the whole-cell extract interacts with γTuRC proteins. Precipitation of U2OS whole-cell extracts with immobilized Abs specific to UFL1$_{301-389}$, C53, or γ-tubulin. Blots were probed with Abs to UFL1, C53, γ-tubulin (γ-Tb), GCP2, GCP4, or calcineurin (Calcin.; negative control). Load (*lane 1*), immobilized Abs without cell extracts (*lane 2*), precipitated proteins (*lane 3*), and Ab-free carriers incubated with cell extracts (*lane 4*). (**F**) Isotype controls. The whole-cell extracts of U2OS were precipitated with immobilized rabbit Ab to myosin or mouse mAb to MAP2 (IgG2b). Blots were probed with Abs to UFL1, C53, γ-tubulin (γ-Tb), GCP2, GCP4, and calcineurin (Calcin.; negative control). Load (*lane 1*), immobilized Abs not incubated with cell extracts (*lane 2*), precipitated proteins (*lane 3*), and carriers without Abs incubated with cell extracts (*lane 4*).

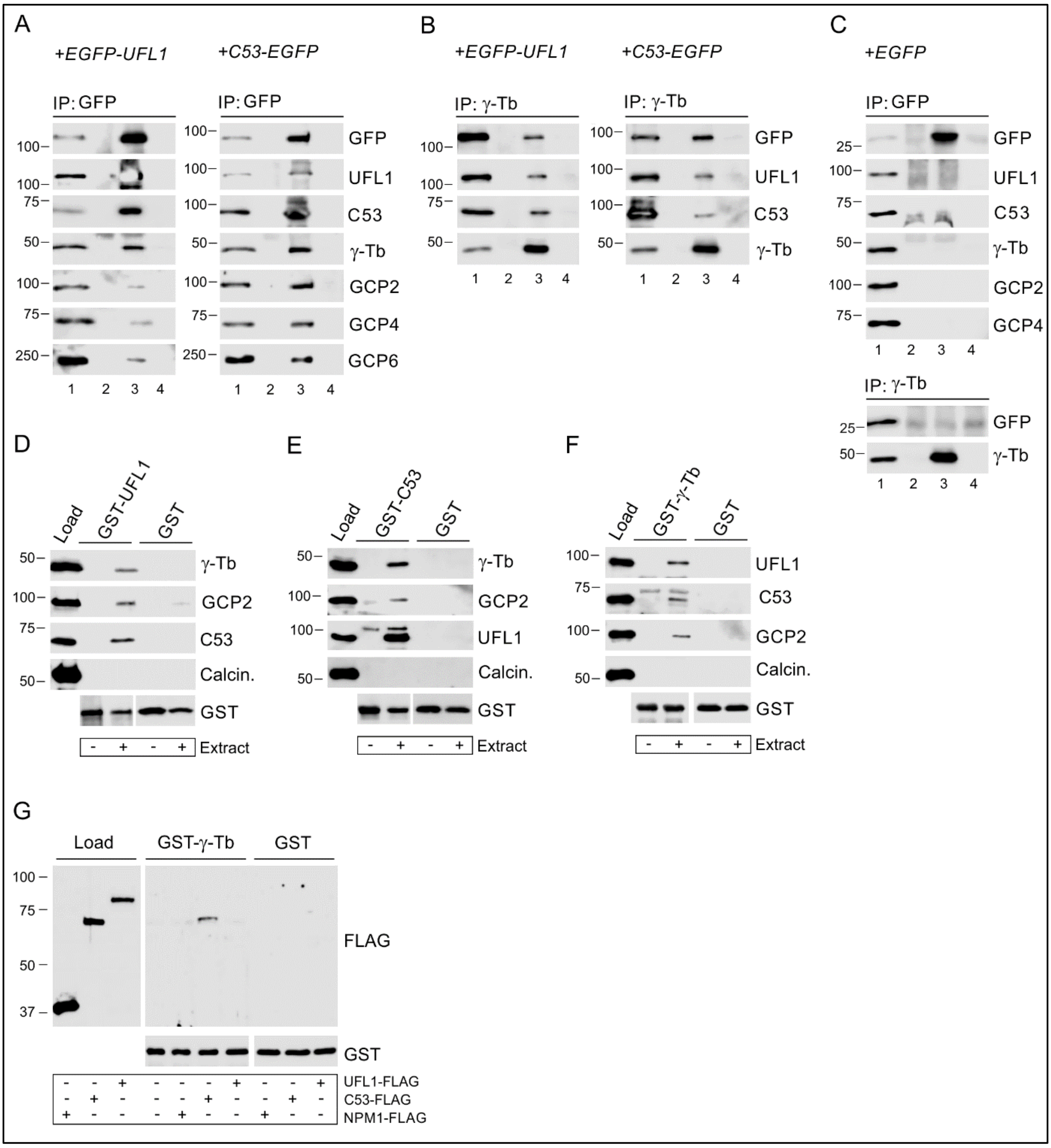

Figure S2. Exogenous UFL1 and C53 interact with γTuRC proteins. (**A-C**) Immunoprecipitation experiments with the whole-cell extracts from U2OS cells expressing EGFP-tagged UFL1 (EGFP-UFL1), C53 (C53-UFL1), or EGFP alone. (**A**) Precipitation with immobilized Ab to GFP. (**B**) Precipitation with immobilized Ab to γ-tubulin. (**C**) Precipitation with immobilized Ab to GFP or γ-tubulin. Blots were probed with Abs to GFP, UFL1, C53, γ-tubulin (γ-Tb), GCP2, GCP4, or GCP6. Load (*lane 1*), immobilized Abs not incubated with cell extracts (*lane 2*), precipitated proteins (*lane 3*), and Ab-free carriers incubated with cell extracts (*lane 4*). (**D-F**) Pull-down assay with GST-tagged UFL1 (**D**), C53 (**E**), and γ-tubulin (**F**). Immobilized GST-fusion proteins (GST-UFL1, GST-C53, GST-γ-tubulin) or immobilized GST alone were incubated with whole-cell extracts from U2OS cells (Load). Blots of bound proteins were probed with Abs to γ-tubulin (γ-Tb), GCP2, C53, UFL1, calcineurin (Calcin.; negative control), and GST. (**G**) Pull-down assay with GST-γ-tubulin and FLAG-tagged proteins. Purified FLAG-tagged UFL1, C53 or nucleophosmin (NPM1; negative control) were incubated with immobilized GST-γ-tubulin or GST alone Immunoblots were probed with Abs to FLAG or GST.

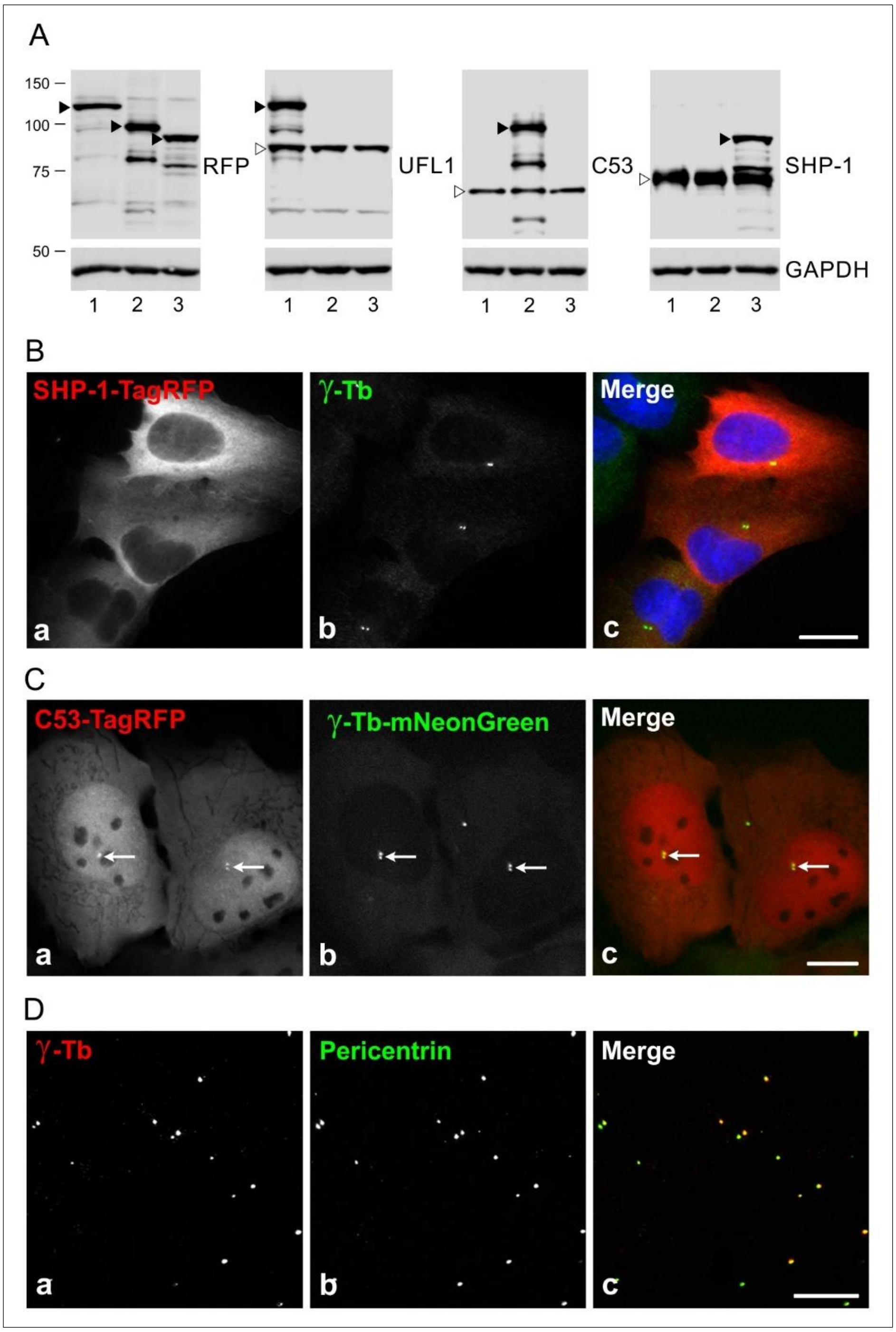

Figure S3. Expression and localization of TagRFP-tagged C53 and SHP-1 in interphase U2OS cells. Immunofluorescence staining of isolated centrosomes. (**A**) Immunoblot analysis of whole-cell lysates from cells expressing UFL1-TagRFP (*lane* 1), C53-TagRFP (*lane 2*), or SHP-1-TagRFP (*lane 3*). Blots were probed with Abs to RFP, UFL1, C53, SHP-1, and GAPDH (loading control). Black and empty arrowheads denote tagged and endogenous proteins, respectively. (**B**) Cells expressing SHP-1-TagRFP (negative control) were fixed and stained with Ab to γ-tubulin. SHP-1-TagRFP (a), γ-tubulin (b; γ-Tb), superposition of images (c, SHP-1-TagRFP, red; γ-tubulin, green; DAPI, blue). Fixation D/F/M. Scale bar, 20 µm. (**C**) Live cell imaging of cells expressing C53-TagRFP and γ-tubulin-mNeonGreen. Localization of C53-TagRFP (a) and γ-tubulin-mNeonGreen (b). Superposition of images (c, C53-TagRFP, red; γ-tubulin-mNeonGreen, green; DAPI, blue). Arrows indicate the same positions. Scale bar, 10 µm. (**D**) Centrosomes isolated by sucrose gradient centrifugation were pelleted on a coverslip, fixed and double-label stained with Abs to γ-tubulin and pericentrin. γ-Tubulin (a; γ-Tb), pericentrin (b), superposition of images (c, γ-tubulin, red; pericentrin, green). Fixation methanol. Scale bar, 20 µm.

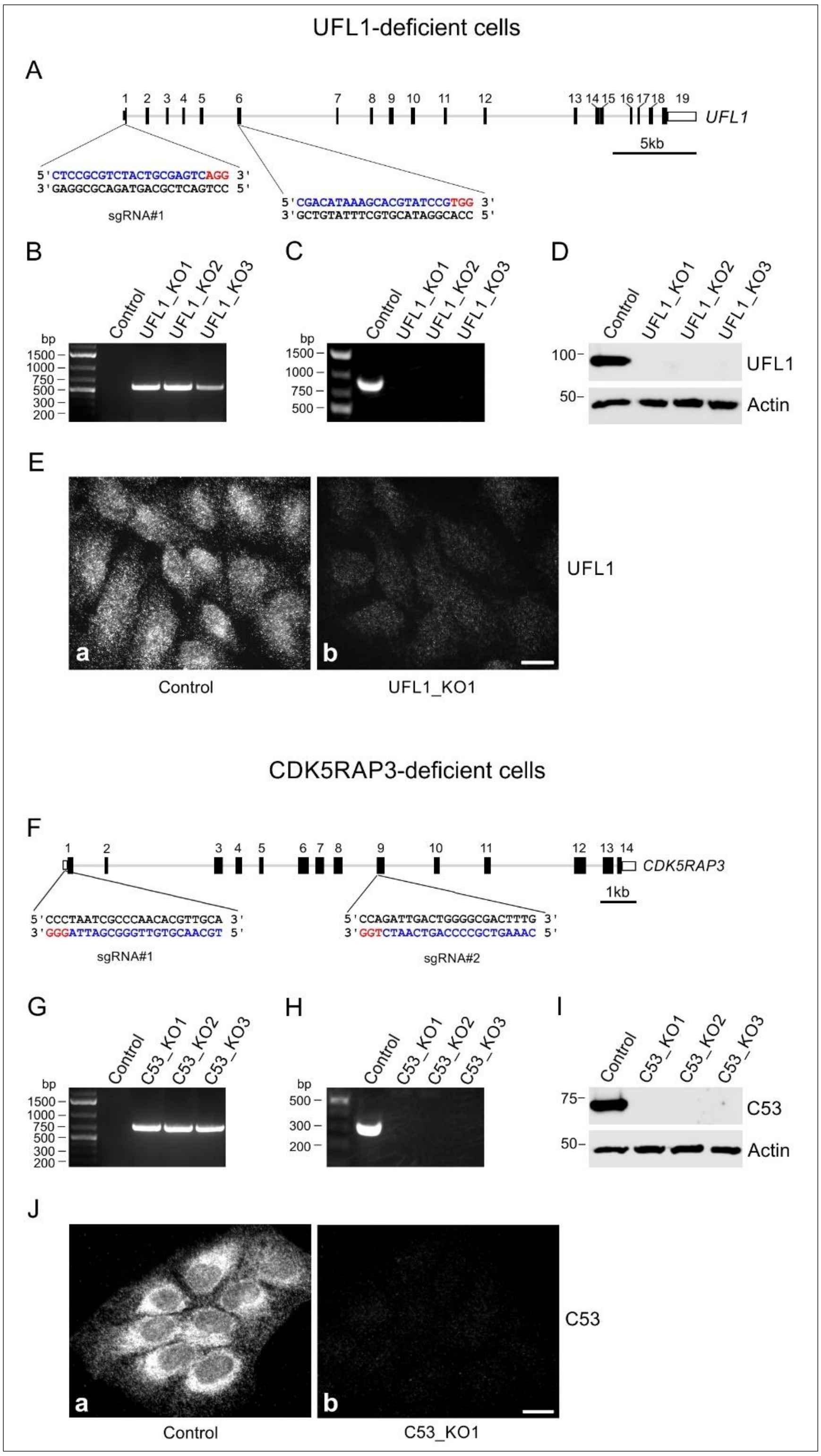

Figure S4. Generation of *UFL1* and *CDK5RAP3* knockout cell lines. (**A-E**) UFL1-deficient cells. (**A**) Schematic diagram of the longest transcript of *UFL1* gene (34.9 kb), containing 19 exons, with sites targeted by guide RNA (sgRNA) sequences. Targeted sites (blue) and protospacer adjacent motifs (PAM; red) are depicted. (**B**) PCR amplification of genomic DNA from control U2OS cells (Control) and UFL1-deficient U2OS cell lines (UFL1_KO1, UFL1_KO2, UFL2_KO3) with primers flanking the deleted region. Due to the large size of the deleted region (~6.8kb), no amplification was found in control cells. Amplification of fragments (~560bp) was detected in UFL1-deficient clones. (**C**) PCR amplification of genomic DNA from control U2OS cells (Control) and UFL1-deficient U2OS cell lines (UFL1_KO1, UFL1_KO2, UFL2_KO3) with primers directed to deleted region. Amplification of fragment (875bp) was detected only in control cells. **(D)** UFL1 protein levels in control and UFL1-deficient U2OS

cell lines analyzed by immunoblotting of whole-cell lysates. Actin served as the loading control. (**E**) UFL1 protein levels in control (a) and UFL1_KO1 (**b**) cells analyzed by immunofluorescence microscopy with Ab to $UFL1_{438-793}$. Fixation F/Tx. The pairs of images were collected and processed in the same manner. Scale bar, 20 μm. (**F-H**) CDK5RAP3-deficient cells. (**F**) Schematic diagram of the longest transcript of *CDK5RAP3* gene (15.2 kb), containing 14 exons, with sites targeted by guide RNA (sgRNA) sequences. Targeted sites (blue) and protospacer adjacent motifs (PAM; red) are depicted. (**G**) PCR amplification of genomic DNA from control U2OS cells (Control) and CDK5RAP3 (C53)-deficient U2OS cell lines (C53_KO1, C53_KO2, C53_KO3) with primers flanking the deleted region. Due to the large size of the deleted region (~6.1 kb), no amplification was found in control cells. Amplification of fragments (~720bp) was detected in C53-deficient clones. (**H**) PCR amplification of genomic DNA from control U2OS cells (Control) and CDK5RAP3 (C53)-deficient U2OS cell lines (C53_KO1, C53_KO2, C53_KO3) with primers directed to the deleted region. Amplification of fragments (302bp) was detected only in control cells. (**I**) C53 protein levels in control and C53-deficient U2OS cell lines analyzed by immunoblotting of whole-cell lysates. Actin served as the loading control. (**J**) C53 protein levels in control (**a**) and C53_KO1 (**b**) cells analyzed by immunofluorescence microscopy with Ab to C53. Fixation F/Tx. The pairs of images were collected and processed in the same manner. Scale bar, 20 μm.

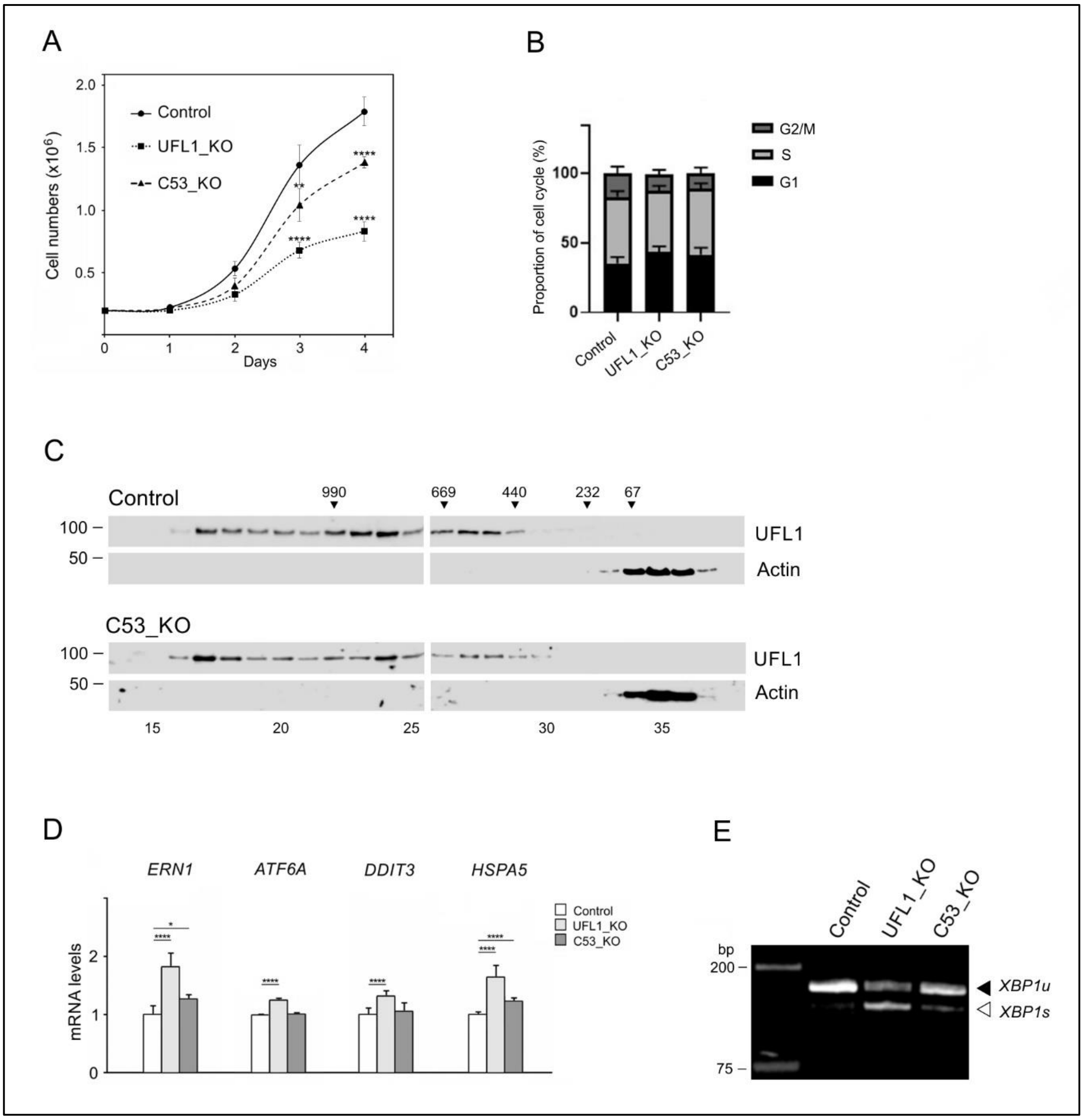

Figure S5. Phenotypes of *UFL1* and *CDK5RAP3* knockout cells. (**A)** Growth curves in control, UFL1_KO, or C53_KO cells. A total of 2 x 10^5 cells was plated for each cell line. The values indicate mean ± SD (n=5). A two-tailed, unpaired Student's *t* test was performed to determine statistical significance. (**B**) Cell cycle analysis of control, UFL1_KO and C53_KO cells. The values indicate mean ± SD (n=3). (**C**) The size distribution of UFL1 and actin in control and C53_KO whole-cell extracts fractionated on the Superose 6 Increase column. The calibration standards (in kDa) are indicated on the top. The numbers at the bottom denote individual fractions .(**D**) Transcription of IRE1α (*ERN1*), ATF6α (*ATF6A*), CHOP (*DDIT3*) and Grp78/BIP (*HSPA5*) genes in UFL1_KO and C53_KO cells relative to the levels in control cells. Data represent the mean ± SD (n=3). One-way ANOVA with Dunnett's multiple comparisons test was performed to determine statistical significance. (**E**) Levels of XBP1 mRNA splicing, determined in total cDNA by RT-PCR, in control, UFL1_KO and C53_KO cells. *, $p < 0.05$; **, $p < 0.01$; ****, $p < 0.0001$.

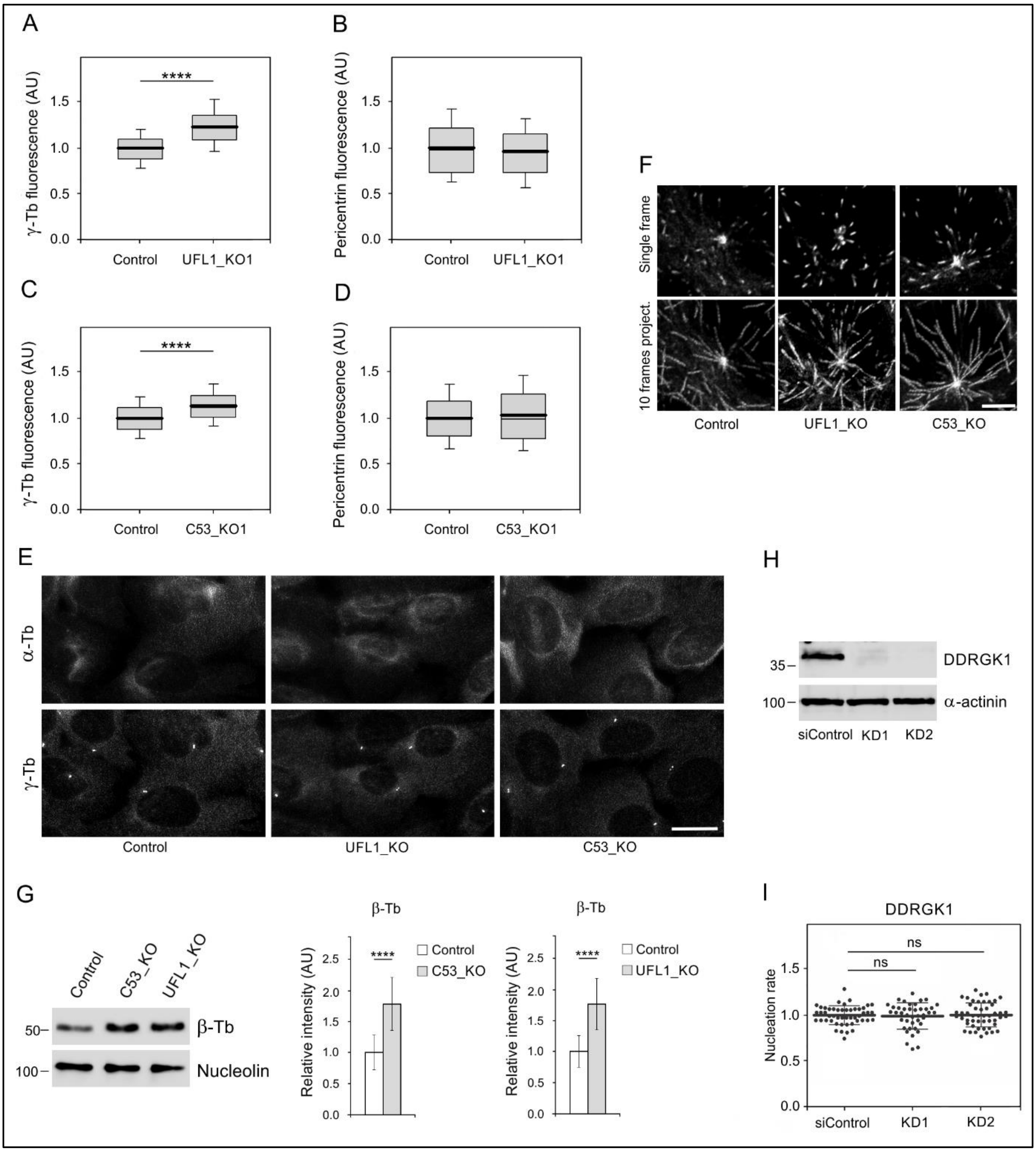

Figure S6. Deletion of UFL1 or C53 stimulates accumulation of γ-tubulin but not pericentrin at the centrosome, enhances microtubule nucleation and the amount of polymerized tubulin. Controls for microtubule regrowth experiments. Depletion of DDRGK1 does not affect microtubule nucleation. (**A-D**) The distributions of γ-tubulin or pericentrin fluorescence intensities (arbitrary units [AU]) in 2-μm ROI at 2.0 min of microtubule regrowth in control, UFL1-deficient (UFL1_KO1) or C53-deficient (C53_KO1) cells are shown as box plots (three experiments for UFL1-KO1 and four experiments for C53_KO1, > 49 cells counted for each experimental condition). (**A-B**) Box plot of γ-tubulin (**A**) and pericentrin (**B**) fluorescence intensities in UFL1_KO1 cells (n=234) relative to control cells (Control, n=247). (**C-D**) Box plot of γ-tubulin (**C**) and pericentrin (**D**) fluorescence intensities in C53_KO1 cells (n=358) relative to control cells (Control, n=322). The bold and thin lines within the box represent mean and median (the 50th percentile), respectively. The bottom and top of the box represent the 25th and 75th percentiles. Whiskers below and above the box indicate the 10th and 90th percentiles. (**E**) Control, UFL1_KO, and C53_KO cells were fixed in the presence of nocodazole and stained for α-tubulin and γ-tubulin. Fixation F/Tx/M. Scale bar, 20 μm. **(F)** Time-lapse imaging of control and UFL1_KO1 or C53_KO1 cells expressing EB3-mNeonGreen. Still images of EB3 (Single frame) and tracks of EB3 comets over 10 s (10 frames project.). Scale bars, 5 μm. (**G**) Immunoblot analysis of

microtubule polymer. Control, UFL1_KO, and C53_KO cells were extracted with 0.2% Triton X-100 at 37°C in MES buffer, and detergent-insoluble fractions were analyzed by immunoblotting with Abs to β-tubulin and nuclear protein nucleolin (loading control). Densitometric quantification of immunoblots is shown on the right. Relative intensities of β-tubulin normalized to control cells and the amount of nucleolin in individual samples. Values indicate mean ± SD (n=12 for UFL1_KO; n=12 for C53_KO). (H) Immunoblot analysis of cells with reduced levels of DDRGK1. Whole-cell lysates from cells transfected with scrambled siRNA (siControl) or DDRGK1 siRNAs (KD1, KD2). Blots probed with Abs to DDRGK1 and α-actinin (loading control). (I) Microtubule nucleation rate (EB3 comets/min) in cells with depleted levels of DDRGK1 (KD1, KD2) relative to control cells transfected with scrambled siRNA (siControl). Three independent experiments (at least 11 cells counted in each experiment). Control (n=49), KD1 (n=41), KD2 (n=49). The bold and thin lines within the dot plot represent mean ± SD. (**A-D, G**) A two-tailed, unpaired Student's *t* test was performed to determine statistical significance. (**I**) One-way ANOVA with Dunnett's multiple comparisons test was performed to determine statistical significance. ****, $p < 0.0001$.

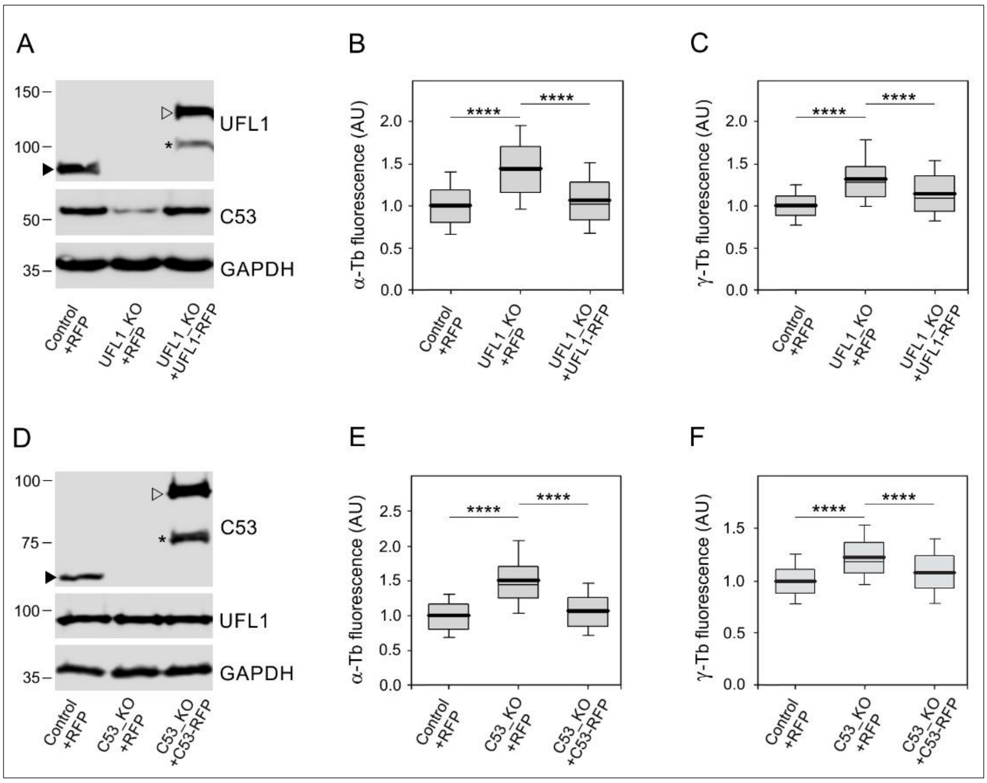

Figure S7. Phenotypic rescue of increased microtubule nucleation in *UFL1* and *CDK5RAP3* knockout cells. (**A**) Immunoblot analysis of UFL1 and C53 in whole-cell lysates from control cells expressing TagRFP (Control+RFP), UFL1_KO cells expressing TagRFP (UFL1_KO+RFP), and UFL1_KO cells rescued by UFL1-TagRFP (UFL1_KO+UFL1-RFP). GAPDH served as the loading control. Black and empty arrowheads and asterisk denote, respectively, endogenous UFL1, UFL1-TagRFP, and its fragment. (**B-C**) The distributions of α-tubulin or γ-tubulin fluorescence intensities (arbitrary units [AU]) in 2-μm ROI at 2.0 min of microtubule regrowth are shown as box plots (four independent experiments, > 30 cells counted for each experimental condition). Box plot of α-tubulin (**B**) and γ-tubulin (**C**) fluorescence intensities in UFL1_KO+RFP (n=181) and UFL1_KO+UFL1-RFP cells (n=298) relative to control cells (Control+RFP, n=267). (**D**) Immunoblot analysis of C53 and UFL1 in whole-cell lysates from control cells expressing TagRFP (Control+RFP), C53_KO cells expressing TagRFP (C53_KO+RFP), and C53_KO cells rescued by C53-TagRFP (C53_KO+C53-RFP). GAPDH served as the loading control. Black and empty arrowheads and asterisk denote, respectively, endogenous C53, C53-TagRFP, and its fragment. (**E-F**) The distributions of α-tubulin or γ-tubulin fluorescence intensities (arbitrary units [AU]) in 2-μm ROI at 2.0 min of microtubule regrowth are shown as box plots (three independent experiments, > 30 cells counted for each experimental condition). Box plot of α-tubulin (**E**) and γ-tubulin (**F**) fluorescence intensities in C53_KO+RFP (n=191) and C53_KO+C53-RFP cells (n=248) relative to control cells (Control+RFP, n=179). (**B, C, E, F**) Bold and thin lines within the box represent mean and median (the 50th percentile), respectively. The bottom and top of the box represent the 25th and 75th percentiles. Whiskers below and above the box indicate the 10th and 90th percentiles. One-way ANOVA with Sidak's multiple comparisons test was performed to determine statistical significance. ****, $p < 0.0001$.

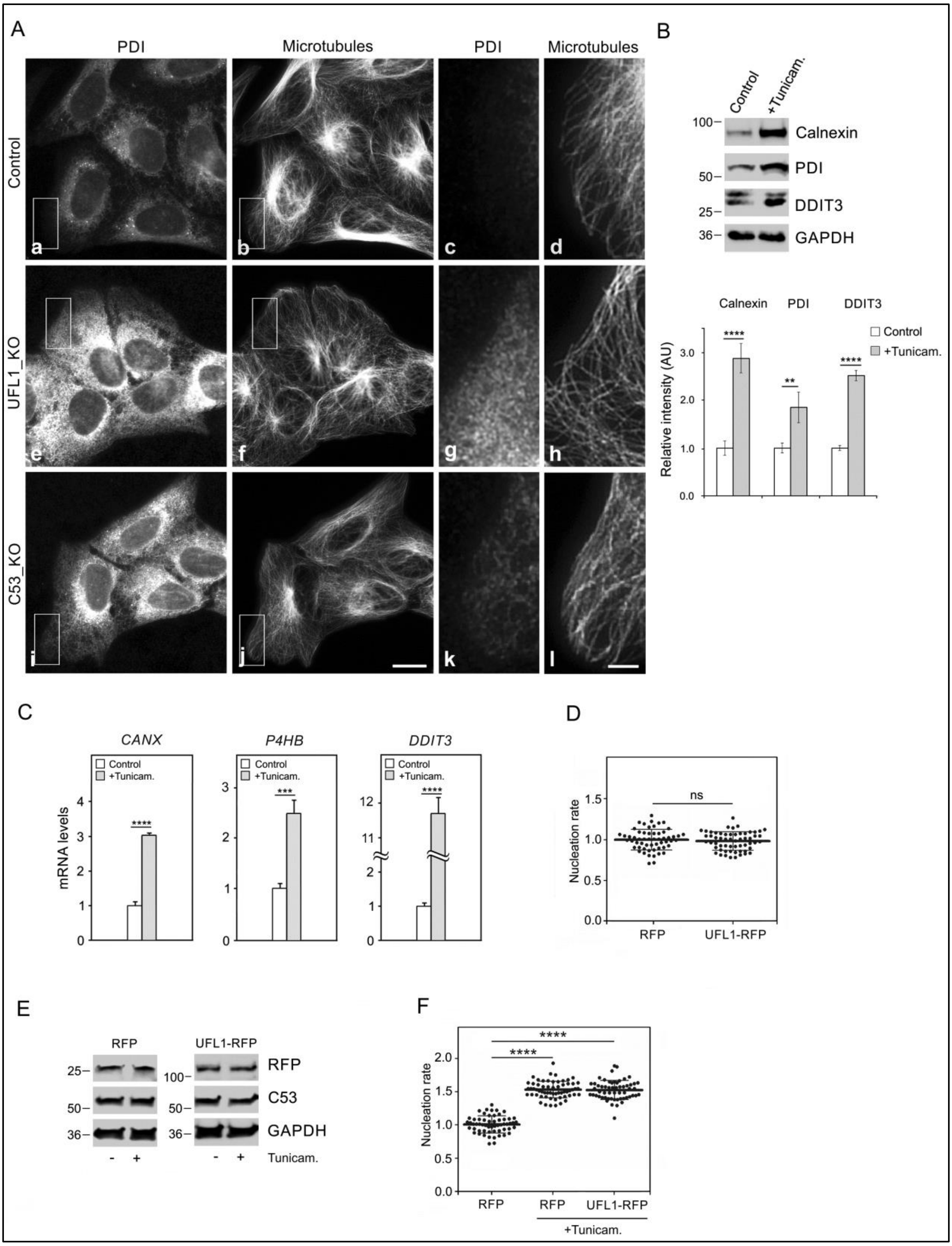

Figure S8. Deletion of UFL1 or C53 induces subcellular redistribution of PDI. Tunicamycin causes upregulation of ER stress-associated proteins and increases centrosomal microtubule nucleation. (**A**) Immunofluorescence microscopy. (**a-d**) Control, (**e-h**) UFL1-deficient (UFL1_KO) and (**i-l**) C53-deficient (C53_KO) U2OS cells. Cells were fixed and double-labeled for PDI (**a, e, i**) and β-tubulin (**b, f, j**; Microtubules). Higher magnification views of the regions delimited by rectangles are shown on the right of images from control (**c-d**), UFL1_KO (**g-h**), and C53_KO cells (**k-l**). Images (**a, e, i**) and (**c, g, k**) were collected and processed in exactly the same manner. Fixation F/Tx. Scale bars, 20 μm (**j**) and 5 μm (**l**). (**B-C, E-F)** U2OS cells were treated with 1 μg/mL tunicamycin (+Tunicam.) or DMSO

carrier (Control) for 24 h. (**B**) Immunoblot analysis of calnexin, PDI, and DDIT3 in whole-cell lysates from control and tunicamycin-treated cells. GAPDH served as the loading control. Densitometric quantification of immunoblots is shown on the right. Relative intensities of corresponding proteins normalized to control cells and the amount of GAPDH in individual samples. Values indicate mean ± SD (n=5 for calnexin; n=4 for PDI; n=3 for DDIT3). (**C**) Transcription of calnexin (*CANX*), PDI (*P4HB*), and DDIT3 (*DDIT3*) genes in cells treated with tunicamycin relative to the levels in untreated control cells. Data represent the mean ± SD (n=3). (**D**) Microtubule nucleation rate (EB3 comets/min) in cells expressing UFL1-TagRFP (UFL1-RFP) relative to control cells (RFP). Three independent experiments (at least 15 cells counted in each experiment). RFP (n=57), UFL1-RFP (n=58). The bold and thin lines within the dot plot represent mean ± SD. (**E**) Immunoblot analysis of cells expressing RFP (control) or UFL1-RFP and treated or not with tunicamycin. Whole-cell lysates probed with Abs to RFP, C53 and GAPDH (loading control). (**F**) Microtubule nucleation rate (EB3 comets/min) in cells treated with tunicamycin (+Tunicam.; RFP; UFL1-RFP) relative to control untreated cells (RFP). Three independent experiments (at least 15 cells counted in each experiment). RFP (n=57), RFP+Tunicam. (n=60), UFL1-RFP+Tunicam. (n=57). The bold and thin lines within the dot plot represent mean ± SD. (**B-D**) Two-tailed, unpaired Student's *t* test was performed to determine statistical significance. (**F**) One-way ANOVA with Dunnett's multiple comparisons test was performed to determine statistical significance. **, $p < 0.01$; ***, $p < 0.001$; ****, $p < 0.0001$.
